# Effectiveness of Psychological Treatments for Problematic Use of Internet, Video Games, Social Media and Instant Messaging: A Systematic Review and Meta-Analysis

**DOI:** 10.3390/ijerph22101598

**Published:** 2025-10-21

**Authors:** Mateo Pérez-Wiesner, Kora-Mareen Bühler, José Antonio López-Moreno, Maria Dolores López-Salmerón

**Affiliations:** 1 Department of Psychobiology and Methodology in Behavioral Sciences, Faculty of Psychology, Somosaguas Campus, Complutense University of Madrid, 28223 Madrid, Spain; kobuhler@ucm.es; 2 Department of Psychology, Faculty of Health Sciences—HM Hospitals, University Camilo José Cela, 28692 Madrid, Spain; mariadolores.lopez.s@udima.es; 3 HM Hospitals Health Research Institute, 28015 Madrid, Spain; 4 MIDELOY Research-Madrid, 28922 Madrid, Spain; 5 Faculty of Psychology and Health Sciences, Distance University of Madrid, 28400 Madrid, Spain

**Keywords:** psychological treatments, problematic use, video games, Internet, Internet Gaming, social media, cognitive behavioral therapy

## Abstract

Adolescence is a developmental stage characterized by increased vulnerability to technology use. Several models have been proposed to explain the psychological processes involved in addictive use. In response to this evidence, therapeutic and preventive intervention programs aim to reduce key symptoms in order to promote health and protect adolescents. This study presents a systematic review and meta-analysis on the effectiveness of psychological therapeutic and preventive interventions for problematic use of Information and Communication Technologies (ICTs) in adolescents (aged 10–21). A total of nine studies (five RCTs and four non-RCTs) with 744 participants were analyzed. The search was conducted following PRISMA guidelines and using the PICO framework. Included studies involved face-to-face or online psychological therapeutic and preventive interventions targeting adolescents, with a particular focus on cognitive behavioral therapy (CBT). Results indicate significant effects in favor of the experimental group in reducing symptoms associated with Internet, video game, social media, and instant messaging addiction, with pooled effect sizes of SMD = −1.53 (RCTs) and SMD = −1.13 (non-RCTs). Despite heterogeneity and potential publication bias, the evidence supports the effectiveness of these interventions, particularly CBT, family therapy, and executive function training. A multidisciplinary approach, early detection, and treatment personalization are recommended. Methodological limitations were identified, highlighting the need for more rigorous future research with attention to gender differences and cultural adaptation.

## 1. Introduction

Information and Communication Technologies (ICTs) have gained central relevance in contemporary society, exerting a transversal impact on education, the economy, healthcare, and daily life [1]. Their prevalence is particularly notable among adolescents and young adults: more than 96% of adolescents own a mobile phone, 98% regularly access the Internet (mainly for social or recreational purposes), and nearly all adolescents and young adults (98.5%) are registered on at least one social media platform, with 83.5% using three or more. In terms of video games, 71.1% of this population spend their free time gaming online, while 99% use at least one instant messaging application [2]. This widespread access has transformed how individuals interact, learn, and work, enabling instant communication, global access to information, and process automation [3]. Among the most notable benefits of ICTs are the democratization of knowledge through online educational platforms [4,5], improved work management [6]—particularly in the business context—and financial and social inclusion, enabling participation in previously inaccessible environments [7]. Furthermore, ICTs foster innovation and efficiency across multiple sectors (including governmental systems), contributing to greater competitiveness and quality of life [8]. Therefore, the appropriate use of ICTs represents a key opportunity for personal and collective development in the modern context [9].

Adolescence is a critical developmental stage in relation to ICT use. It begins biologically around the age of 10 and may extend until the age of 18 or even 21, depending on the author consulted [10]. This period is characterized by profound physical, psychological, and social changes, also influenced by genetic and environmental variables. Biologically, it begins with the activation of the hypothalamic–pituitary–gonadal axis and the emergence of secondary sexual characteristics [11]. Nonetheless, there is ongoing debate regarding the age range that defines adolescence. For instance, the World Health Organization (WHO) defines adolescence as the period between 10 and 19 years of age [12], while the American Society of Adolescent Health and Medicine extends this range to 21 years, distinguishing early, middle, and late stages of adolescence [13], and in some cases even up to 24 years of age [14]. This extension may be explained by the lack of synchrony between physical, sexual, psychological, and social maturation, meaning that development is not uniform and depends on multiple individual and contextual factors. Therefore, adolescence should be understood as a gradual and multidimensional process in which the child transitions to adulthood, consolidating identity and autonomy within a context of profound biological and psychosocial transformation [15,16].

During this period, the human brain undergoes significant changes, particularly in the prefrontal regions and limbic system, which are responsible for motivation, reward-seeking, and behavioral inhibition [17,18]. This neurobiological imbalance represents a phase of heightened vulnerability to the development of addictive behaviors, including both substance use and problematic or behavioural addictions related to ICTs, due to the immaturity of control mechanisms and the predominance of reward systems [2,19].

It is important to note that early substance use or problematic ICT use during adolescence is associated with a higher risk of developing addictions and more severe clinical problems later in life [20]. Additionally, the consolidation of addictive habits during this period can lead to adverse psychological, social, and academic outcomes, including an increased risk of academic failure, interpersonal and family problems, and socioemotional disturbances [21]. Given that adolescents represent 16% of the global population, it is essential to support their development and well-being to ensure healthier adults [22], thereby reducing the risk of chronic diseases, mental disorders, and risky behaviors that may compromise quality of life and societal contribution [21,23].

Problematic use of ICTs mirrors behavioral patterns observed in substance use disorders, such as dependency, loss of control, positive and negative reinforcement, withdrawal symptoms, and even shared neurobiological mechanisms [24].

In the present study, the term “problematic use” of ICTs is employed as a descriptive category encompassing various digital behaviors characterized by loss of control, functional impairment, and psychological distress. However, we acknowledge the conceptual distinction between formally recognised disorders in the International Classification of Diseases [25], such as Gaming Disorder, and other dysfunctional usage patterns (e.g., problematic use of social media or instant messaging) for which consensual diagnostic criteria have not yet been established [26,27]. This differentiation enables the interpretation of results from a dimensional perspective, consistent with the heterogeneity of the studies included in this review.

Among the various ICT-related issues, there is a growing concern about Problematic Internet Use (PIU) [26]. This term refers to excessive online activity associated with functional impairment and clinical distress, including gaming, gambling, online shopping, pornography consumption, social media use, cyberchondria, digital hoarding, cyberbullying, and excessive streaming, often exhibiting addictive, impulsive, and/or compulsive characteristics [25,28]. The WHO has created a diagnostic category for “other specified or unspecified disorders due to addictive behaviors”, allowing such behaviors to be diagnosed if clinical criteria are met [25]. Intrapersonal factors such as genetics and personality, as well as social and environmental elements, help explain the origins of PIU. For instance, variations in dopaminergic and serotonergic systems, gene–environment interactions, or personality traits such as low conscientiousness and agreeableness, combined with high levels of neuroticism and extraversion, are associated with increased risk. Impulsivity and low self-esteem also play a significant role. Moreover, the quality of social relationships, both familial and peer-related, has been shown to influence screen time [29,30]. In 2017, Anderson [30] concluded that PIU arises from the interplay of individual, contextual, and activity-related factors that may lead adolescents to engage in problematic online behavior.

These findings align with various addiction models. For example, the Bioecological Model of Human Development (BMHD) by Bronfenbrenner & Morris (2006) explains how human behavior evolves due to the dynamic interaction of individual and contextual factors over time [31]. In relation to ICTs, the Internet Addiction Model (IAM) by Douglas et al. (2008) [32] identifies “push” and “pull” factors influencing Internet use. Push factors include aspects that meet individual needs and motivations, such as avoiding face-to-face interaction or escaping reality. Pull factors refer to Internet attributes that make it potentially addictive, such as low cost, relief from social isolation, ease of communication, convenience, and anonymity, all of which shape an individual’s propensity for excessive use [32]. More recently, the I-PACE model (Interaction of Person–Affect–Cognition–Execution) by Brand et al. [33], proposes that cognitive biases and coping styles related to Internet use moderate the associations between predisposing factors and PIU features, as well as related psychopathologies and personality traits. These processes are reinforced by positive and negative reinforcement mechanisms, which reduce inhibitory control and increase Internet use. Despite several studies and explanatory models on PIU, pathological gaming, and substance addiction, further research is needed to clarify overlaps and distinctions among these psychological constructs [34].

However, in a systematic review by Tunney et al. [35] on PIU models, the authors identified predisposing factors (e.g., mental health issues, personality, temperament, neuropsychological profile, attachment, and early experiences); precipitating factors (e.g., Internet features, motivations for use, mood regulation, conditioning and habit formation, life stress, and lapses in self-regulation); and maintenance factors (e.g., Internet characteristics, cognitive and emotional regulation processes, socialization, conditioning, poor self-regulation, and conflict). Protective factors, such as social support, level of online engagement, and Internet self-efficacy, were also highlighted [35].

Based on these models, the main psychological therapeutic and preventive interventions for behavioral addictions in adolescents include psychoeducation, cognitive behavioral therapy (CBT), motivational interventions aimed at enhancing treatment engagement, mindfulness-based therapies, and third-wave approaches, which are less empirically supported and therefore recommended in combination with other techniques. These can be delivered face-to-face or online, individually or in groups [27,36]. Specifically, CBT remains the most effective intervention for online gaming addiction, as demonstrated by the PIPAC program [37,38]. For Internet addiction, the most effective treatments according to Li et al. [39] were CBT, motivational interviewing, mindfulness-based interventions, family therapy, and multilevel interventions. For excessive use of instant messaging applications, treatment is typically addressed within broader programs targeting technology-related addictions [40].

Nevertheless, addressing behavioral addictions requires a multidisciplinary approach that considers the complexity of contributing factors and emphasizes the need for personalized therapeutic and preventive interventions [41,42,43]. The present study aims to conduct a systematic review and meta-analysis evaluating the effectiveness of psychological therapeutic and preventive interventions for adolescents aged 10–21 years in addressing the problematic use of video games, social media, the Internet, and instant messaging. The specific objectives are as follows:

(1) To identify and analyze the psychological, therapeutic, and preventive interventions implemented with adolescents to address problematic use of video games, online gaming, social media, and instant messaging.
(2) To evaluate the effectiveness of cognitive behavioral interventions in reducing symptoms associated with problematic ICT use.
(3) To compare the magnitude of effects of psychological therapeutic and preventive interventions relative to control groups.
(4) To synthesize available evidence to establish evidence-based recommendations for clinical practice and future research focused on the prevention and treatment of problematic use of video games, social media, and instant messaging among adolescents.

## 2. Materials and Methods

The protocol for this systematic review and meta-analysis was developed and registered in the International Prospective Register of Ongoing Systematic Reviews, York, United Kingdom (PROSPERO) under registration number CRD420251029371. It was prepared in accordance with the PRISMA (Preferred Reporting Items for Systematic Reviews and Meta-Analyses) statement and checklist (Supplementary Material Table S4), following the guidelines by Page et al. [20].

### 2.1. Search Strategy

The literature search was conducted in the following databases: MEDLINE (PubMed), PsycINFO, SCOPUS, and Web of Science (WOS). Keywords focused on psychological therapeutic and preventive interventions, problematic use/addiction/excessive use of video games, social media, and instant messaging. Keyword development involved the use of MESH terms, truncations, and Boolean operators (AND and OR). A subsequent review of the search strategy was requested from the librarians at Camilo José Cela University (UCJC). The final search terms were: (Phychotherap * OR “mental health treatm *” OR “phychological treatm *” OR “Behavioral therap *”) AND (“problematic use” OR addiction * OR disorders OR abuse OR “excessive use”) AND (game * OR gaming OR “video game *” OR “computer game *” OR “social media” OR “social network *” OR “instant message *”) AND (Adolescent * OR youth * OR teen * OR child *).

Additionally, the PICO framework (Population, Intervention, Comparison, and Outcome) was applied. The Population (P) included adolescents aged 10 to 21 years presenting symptoms or disorders associated with problematic ICT use (Internet, video games, online gaming, or social media), assessed using validated self-report instruments. The Intervention (I) encompassed psychological therapeutic and preventive programs delivered either individually or in groups. The Comparison (C) group consisted of adolescents receiving alternative interventions such as treatment-as-usual, waitlist conditions, pharmacotherapy, or participation in randomized controlled trials (RCTs) or quasi-experimental studies (non-RCTs).

### 2.2. Study Selection, Quality and Risk of Bias Assessment

**Figure 1:**
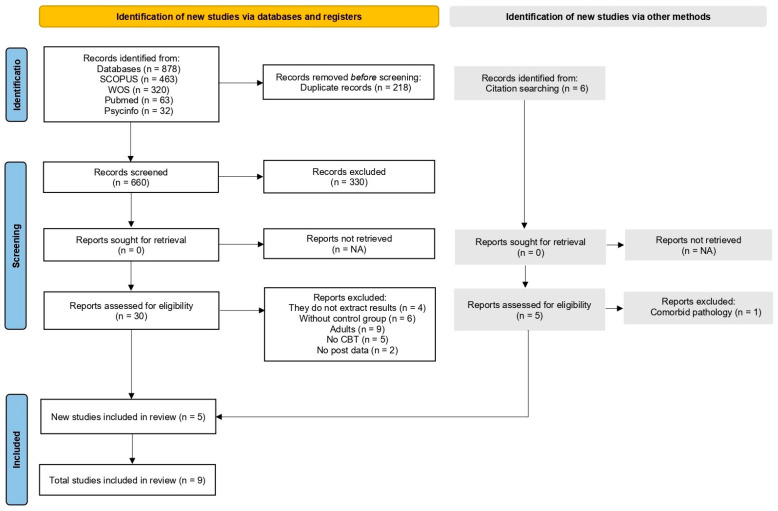
PRISMA Flowchart.

Study selection for this review was conducted using Rayyan (Rayyan Systems Inc., Cambridge, MA, USA, https://www.rayyan.ai (accessed 21 February 2025)), a platform designed to streamline the screening process in systematic reviews [44]. A total of 878 articles were initially identified through a parallel and blinded screening by two reviewers, who removed 218 duplicates. Following title and abstract screening, 30 studies were retained for full-text review. Subsequently, M.P. and M.D.L. independently reviewed the full texts, resolving discrepancies with the participation of a third reviewer (J.A.L.). A manual search of the scientific literature was also conducted to identify any additional relevant studies not captured in the initial search. The final selection comprised nine studies, as illustrated in the PRISMA flow diagram (Figure 1) [45].

Strict inclusion and exclusion criteria were established to ensure the relevance and methodological quality of the selected studies. Inclusion criteria encompassed studies published in the last five years, written in English, addressing face-to-face or online psychological therapeutic and preventive interventions targeting adolescents aged 10 to 21. Both individual and group therapies aimed at treating Internet use, video game, online gaming, or social media problems were included. Eligible studies were required to include a control group (no treatment or placebo), employ standardized assessment instruments, and focus on psychological interventions. Excluded studies comprised non-original works (e.g., book chapters, theses, undergraduate or master’s dissertations), unpublished studies (e.g., abstracts or conference proceedings), and studies exhibiting methodological weaknesses in randomization, blinding, or dropout management. Publications in languages other than English were also excluded, as were systematic reviews, meta-analyses, studies lacking pre- and post-intervention assessments, those involving comorbidities or dual diagnoses, or those in which the experimental group did not receive the full intervention or lacked an experimental condition.

The methodological quality of the included studies was assessed using two specific checklists: one for quasi-experimental studies (non-RCTs) and another for randomized controlled trials (RCTs). Risk of bias in RCTs was evaluated using the Revised Cochrane Risk-of-Bias Tool for Randomized Trials (RoB 2) [46], whereas the Risk of Bias in Non-Randomized Studies—of Exposures (ROBINS-E) tool was applied to non-RCTs [47]. Two independent reviewers (M.P. and M.D.L.) conducted these assessments in parallel, and discrepancies were resolved with the assistance of a third reviewer (J.A.L.).

### 2.3. Certainty of Evidence (GRADE Approach)

We assessed the certainty of the evidence for each outcome using the Grading of Recommendations, Assessment, Development, and Evaluation (GRADE) framework, in accordance with the Cochrane Handbook for Systematic Reviews of Interventions [48]. The GRADE approach categorizes the certainty (confidence) of the evidence as high, moderate, low, or very low, based on five domains: risk of bias, inconsistency, indirectness, imprecision, and publication bias.

Randomized controlled trials (RCTs) were initially rated as providing high-certainty evidence and could be downgraded by one or two levels per domain if significant limitations were identified. Non-randomized studies were initially rated as low-certainty and could be further downgraded or, in exceptional cases, upgraded (e.g., in the presence of a large effect size or a dose–response relationship).

Risk of bias was assessed using the RoB 2 or ROBINS-E tools applied in this review. Inconsistency was evaluated using heterogeneity statistics (I^2^, τ^2^, and Q-tests) and visual inspection of forest plots. Indirectness was determined by examining the correspondence between the included populations, interventions, comparators, and outcomes, and the specific research question of this review. Imprecision was assessed based on the width of the 95% confidence intervals and the total information size. Publication bias was explored through funnel plot asymmetry and Egger’s test, when applicable.

Two reviewers (M.P. and M.D.L.) independently assessed each domain, with disagreements resolved through discussion or consultation with a third reviewer (J.A.L.). Justifications for all decisions were documented in explanatory footnotes accompanying the Summary of Findings table (Supplementary Material Table S5).

### 2.4. Data Synthesis and Statistical Analysis

A narrative synthesis was provided for all included studies. For the meta-analysis, a random-effects model using the Restricted Maximum Likelihood (REML) estimator was applied. All statistical analyses were conducted using RStudio (version 4.3). RCTs and non-RCTs were analyzed separately to account for methodological and quality differences between study designs. The main outcome assessed was the effect of CBT in reducing addiction-related symptoms or problematic ICT use (Internet, video games, online gaming, social media, and instant messaging) when comparing experimental and control groups, using standardized mean differences (SMDs). Between-study heterogeneity was evaluated using Q-statistics, τ^2^ (estimated total heterogeneity), and I^2^ (with high heterogeneity defined as ≥70%) [49]. Publication bias was assessed using funnel plots, in which standardized mean differences were plotted against the standard error for each study. Results were presented using forest and funnel plots.

To explore potential sources of heterogeneity, mixed-effects meta-regression models were performed with the REML estimator. Cultural context (Eastern vs. Western settings), intervention duration, and delivery modality (individual vs. group) were included as potential moderators. Separate models were estimated for RCTs and non-RCTs. This approach enabled the examination of how study-level characteristics contribute to between-study variability while accounting for within-study sampling error [50].

## 3. Results

### 3.1. Study Selection in the Review

A total of nine studies met the inclusion criteria. Of these, three were conducted in Europe (Spain [51], Germany [52] and Turkey [53]) and six in Asia (two from China [54,55], two from Thailand [56,57], one from South Korea [56] and one from the Philippines [57]). Five studies used a randomized controlled trial (RCT) design [52,53,54,55,58], while four followed a non-randomized quasi-experimental (non-RCT) design [51,56,57,58,59]. The total sample comprised 744 participants aged 11 to 21. Of these, 482 were included in the RCT studies (age range: 11–21) [52,53,54,55,58] and 262 in the non-RCT studies (age range: 12–18) [51,56,57,58,59].

All RCT studies [52,53,54,55,58], implemented CBT-based interventions. Four studies delivered a direct intervention [53,54,55,58] while one [49] addressed both prevention and intervention. Three studies focused specifically on Internet Gaming Disorder (IGD) [54,55,60], one on Internet Addiction (IA) [51], and only Lindenberg et al. (2022) [52] expanded the scope to include both IGD and generalized Internet Use Disorder. The number of sessions ranged from 4 to 16.

Among the non-RCT studies [51,56,57,58,59], three delivered intervention programs [51,56,57] while one included both prevention and intervention [56]. The targeted ICT behaviors varied: two focused on IGD [49,55], one investigated Social Media Addiction (SMA) [54], and Yang & Kim (2018) [59] addressed general Internet Addiction. Sessions ranged from 10 to 22, with an overall average of 10.44 sessions across all studies.

Assessment tools varied by target variable. For IGD, commonly used instruments included the IGDS9-SF (Internet Gaming Disorder Scale–Short Form), based on DSM-5 criteria [59] and the IGD-20 Test (Internet Gaming Disorder Test) [47]. Tools used to assess Internet addiction included the Internet Addiction Scale [51] and the Internet Addiction Proneness Scale [56]. Two studies measured gaming addiction using the GAME-Q (Gaming Motivation Questionnaire) [55], OGAS (Online Gaming Addiction Scale), and GAST (Gaming Addiction Screening Test) [53]. One study assessed addiction symptoms in general [52]. For SMA, Kumkronglek et al. (2023) [56] employed the S-MASS (Muscle Appearance Satisfaction Scale–Short Version), in addition to a life skills test.

Several studies also assessed additional variables, such as impulsivity via the Barratt Impulsiveness Scale [53], personality patterns and clinical syndromes using the MACI (Millon Adolescent Clinical Inventory) [47], emotional intelligence via the TMMS-24 (Trait Meta-Mood Scale, 24 items) [47], and emotional symptoms via the DASS-21 (Depression, Anxiety and Stress Scales, 21 items) [53]. Behavioral and emotional functioning was assessed with the YSR/11–18 [47], and CBCL/6–18 (Youth Self Report for ages 11–18) [47]. Social skills were evaluated using the Social Skills Scale (EHS), and therapeutic engagement using the Working Alliance Theory of Change Inventory (WATOCI) [47]. Only Ji & Wong (2023) [54] assessed motivation, depression, and anxiety, without specifying the instruments used. The full characteristics of the included studies can be found in Supplementary Tables S1 and S2.

### 3.2. Risk of Bias of the Studies

Risk of bias and methodological quality assessment results are presented in Supplementary Table S3. Overall, 33.3% of the studies showed low risk of bias and high methodological quality, 20% of the RCTs [52,53,54,55,59] and 50% of the non-RCTs [51,56,57,58,59]. Meanwhile, 66.7% exhibited some risk of bias (80% of the RCTs and 50% of the non-RCTs). In RCTs [52,55,58], the main concerns stemmed from the lack of clear randomization protocols, ambiguous analytical definitions, and insufficient blinding of therapists and participants. In non-RCTs [51,56,59], the lack of randomization itself hindered control over confounding variables, such as motivation for change or family dynamics. In both types of studies, risk of bias was also associated with a lack of transparency regarding dropouts.

According to the GRADE assessment, the certainty of evidence was *very low* for both RCTs and non-RCTs studies, reflecting the high or unclear risk of bias, substantial heterogeneity, and potential publication bias. A detailed Summary of Findings table is available in Supplementary Material Table S5.

### 3.3. Model of Studies

**Figure 2:**
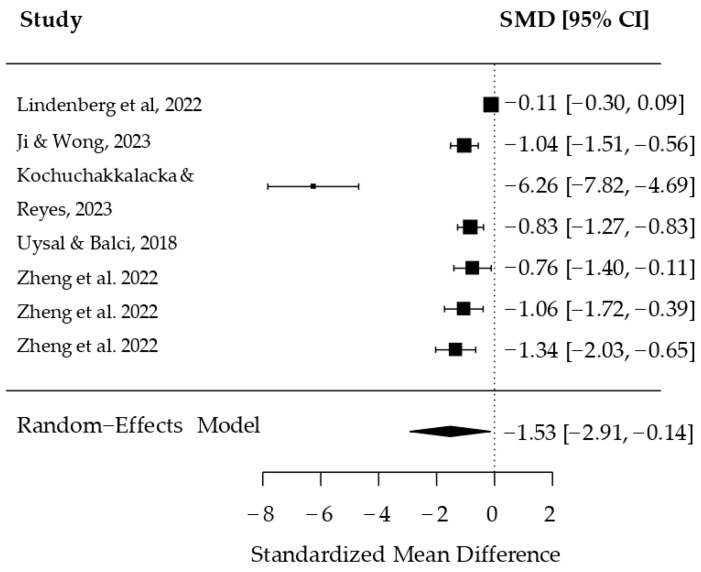
Forest plot of the RCT studies included in the meta-analysis [52,53,54,55,58].

A meta-analysis was performed using a random-effects model, separately for RCT [52,55,58] and non-RCT studies. The RCT analysis included five studies; Zheng et al. (2022) [55] compared three experimental groups to one control group (k = 7; REML estimator). All studies reported standardized mean differences (SMDs) for the primary outcome. Individual and combined effect estimates are displayed in Figure 2 (forest plot).

The estimated tau^2^ value was 3.36 (SE = 2.02), indicating substantial heterogeneity among studies. The square root of tau^2^ (tau) was 1.83. The proportion of variability attributable to true heterogeneity (I^2^) was 98.20%, suggesting considerable variance beyond sampling error. The H^2^ statistic was 55.44, indicating that total variability greatly exceeded what would be expected by chance.

The heterogeneity test yielded a Q value of 83.10 (df = 6, *p* < 0.0001), confirming statistically significant heterogeneity.

The combined effect estimate was an SMD of −1.53 (SE = 0.71, z = −2.16, *p* = 0.0309), with a 95% confidence interval of −2.91 to −0.14. This result indicates a statistically significant overall effect favoring the intervention.

In Figure 2 (forest plot), individual study estimates ranged from −0.11 (95% CI: −0.30, 0.09) [49]) to −6.26 (95% CI: −7.82, −4.69) [59]). Other notable results included Ji & Wong (2023) [50] (SMD = −1.04, 95% CI: −1.51, −0.56), Uysal & Balci (2018) [53] (SMD = −0.83, 95% CI: −1.27, −0.83), and three estimates from Zheng et al. (2022) [55], SMDs of −0.76 (95% CI: −1.40, −0.11), −1.06 (95% CI: −1.72, −0.39), and −1.34 (95% CI: −2.03, −0.65). Additionally, an SMD of −1.53 (95% CI: −2.91, −0.14) was reported for another estimate by Zheng et al. (2022) [55].

**Figure 3:**
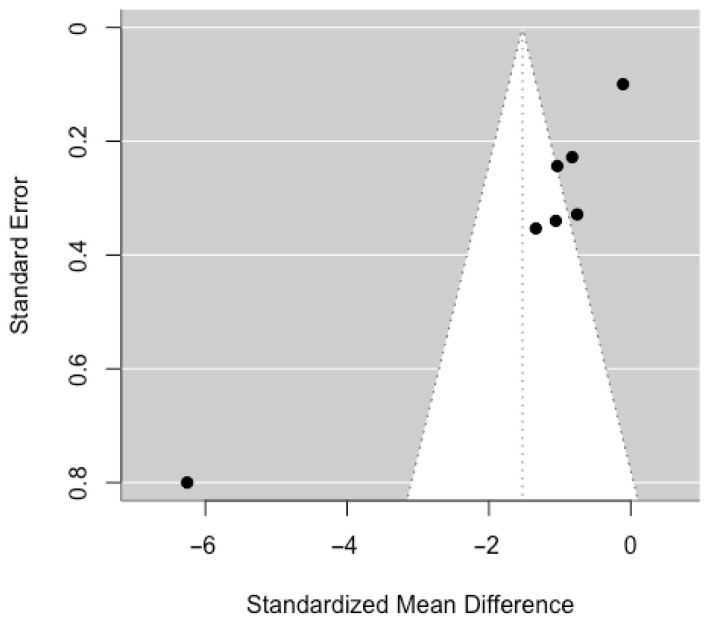
Dispersion of the standardized mean difference (SMD) versus the standard error for the RCT studies included in the meta-analysis.

To assess potential publication bias, a funnel plot was examined (Figure 3). The plot revealed asymmetry, with a predominance of studies reporting negative effects and a lack of studies showing null or positive effects with low standard error. This asymmetry may suggest the presence of publication bias; however, caution is warranted due to the small number of included studies (*n* = 7), which limits the power of funnel plots to detect bias. No formal statistical tests (e.g., Egger’s test or trim-and-fill method) were conducted due to the limited sample size. Nonetheless, the observed asymmetry cannot rule out potential bias and should be considered when interpreting the combined effect.

**Figure 4:**
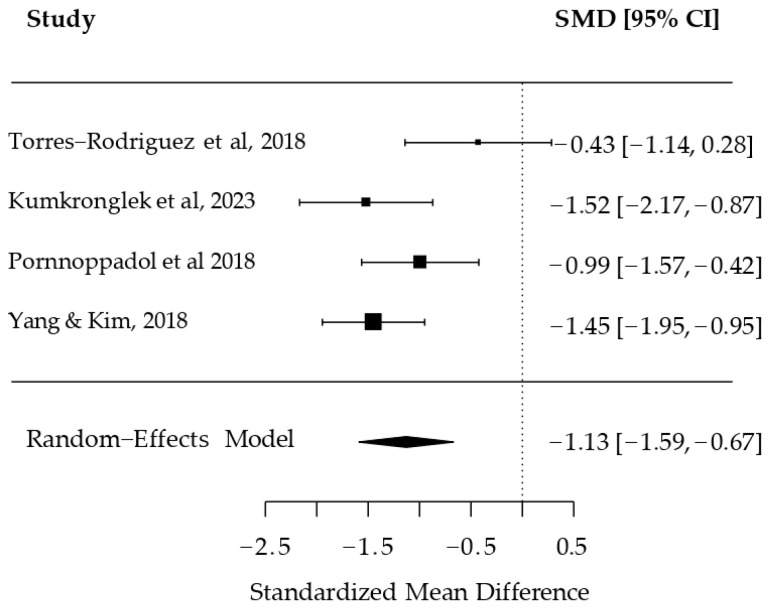
Forest plot of the studies non-RCT included in the meta-analysis [51,56,57,59].

The synthesis of the results of the meta-analysis of the non-RCT studies [51,56,57,58,59] (k = 4; REML estimator), also reported SMDs for the primary outcome. Individual and combined effects are shown in Figure 4.

The estimated tau^2^ was 0.12 (SE = 0.18), suggesting moderate heterogeneity. Tau was 0.35. The I^2^ was 56.72%, indicating that over half the observed variability was due to true differences rather than chance. H^2^ was 2.31.

The Q test for heterogeneity yielded a value of 6.83 (df = 3, *p* = 0.0774), which did not reach statistical significance, though this may reflect the test’s limited power given the small number of studies.

The combined effect estimate was an SMD of −1.13 (SE = 0.23, z = −4.85, *p* < 0.0001), with a 95% confidence interval of −1.59 to −0.67. This result indicates a significant negative effect in favor of the intervention.

In Figure 4 (forest plot), individual effect sizes ranged from −0.43 (95% CI: −1.14, 0.28; [56]) and −1.52 (95% CI: −2.17, −0.87; [55]). Kumkronglek et al. (2023) [56] reported an SMD of −0.99 (95% CI: −1.57, −0.42), while Torres-Rodríguez et al. (2018) [51] reported an SMD of −1.45 (95% CI: −1.95, −0.95). The model-estimated combined effect was −1.13 (95% CI: −1.59, −0.67).

**Figure 5:**
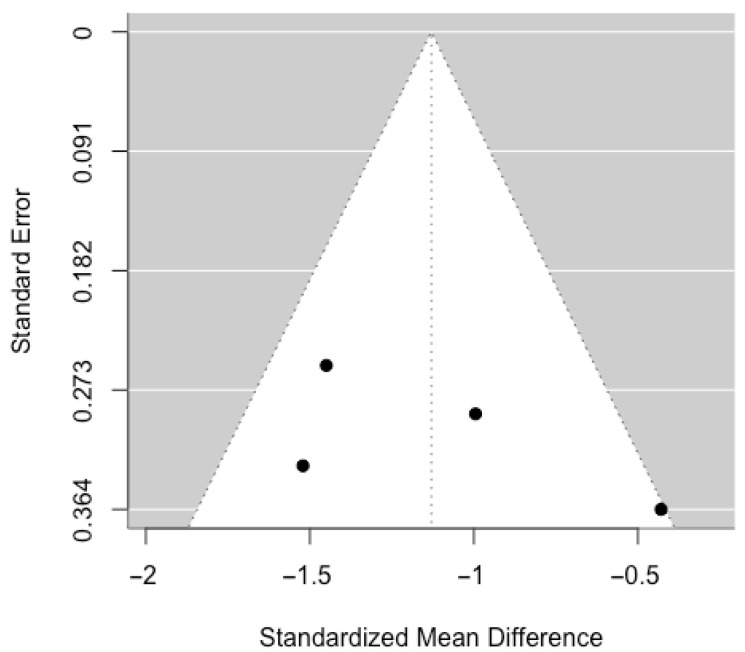
Dispersion of the standardized mean difference (SMD) versus the standard error for the non-RCT studies included in the meta-analysis.

To assess publication bias, the funnel plot (Figure 5) displayed mild asymmetry, with an absence of studies reporting near-zero or positive effects with low standard error. This may suggest publication bias, although the small number of studies (*n* = 4) limits the interpretability of the plot. No formal statistical tests for publication bias were performed due to the limited sample size. Thus, while asymmetry may indicate possible bias, no firm conclusions can be drawn.

Given the substantial heterogeneity observed in both RCTs and non-RCTs (I^2^ = 98.2%), separate metaregressions were conducted to explore potential moderators. For RCTs, the model including cultural context, intervention duration, and delivery modality accounted for 86.2% of the between-study variance (R^2^ = 86.2%), with the overall moderation effect being significant (QM(3) = 19.11, *p* < 0.001). Intervention duration (β = –0.48, *p* < 0.001) and delivery modality (β = 3.71, *p* < 0.001) significantly predicted effect sizes, suggesting that longer and individually delivered interventions were associated with greater efficacy, while cultural context was not significant (*p* = 0.92). In contrast, for non-RCTs, cultural context emerged as a significant moderator (QM(1) = 4.78, *p* = 0.0287), fully explaining the between-study heterogeneity (I^2^ = 0%). Studies conducted in Eastern contexts showed stronger intervention effects (β = –0.91, 95% CI [–1.72, –0.09]) compared with those in Western contexts.

## 4. Discussion

The aim of this study was to conduct a systematic review and meta-analysis evaluating the effectiveness of psychological therapeutic and preventive interventions for ICT-related addictions among adolescents.

Regarding the first specific objective—identify and analyze the types of psychological therapeutic and preventive interventions implemented with adolescents to address Internet, video game, online gaming, and social media addiction—the majority of interventions focused on Internet Gaming Disorder (IGD). In all cases, CBT was integrated with complementary techniques or therapeutic approaches, such as mindfulness or interpersonal skills training [58]. This emphasis on IGD may be attributed to several factors: its inclusion in both the ICD-11 and the revised DSM-5, the established tradition of treatment protocols for pathological gambling, and the theoretical overlap between models of gambling disorder and IGD [58]. No standardized protocol or consensus was identified regarding the optimal number of sessions for adolescent populations; however, the average number was comparable to that of existing substance addiction programs [41].

Concerning the second and third objectives—evaluating the effectiveness of cognitive behavioral interventions in reducing symptoms associated with ICT-related addiction, and comparing the magnitude of treatment effects relative to control groups—the meta-analysis demonstrated that psychological interventions in adolescents have a statistically and clinically significant impact in reducing Internet, video game, and social media addiction. Both RCTs and non-RCTs yielded negative and statistically significant effect sizes in favor of the intervention group, suggesting that these treatments—particularly CBT when combined with other approaches—are effective in reducing symptoms of ICT-related addiction. These findings are consistent with those reported by Alzahrani and Griffiths (2024) [61] and Nagata et al. (2025) [62].

Among the RCTs, the pooled effect size was substantial (SMD = −1.53), although accompanied by high heterogeneity (I^2^ = 98.2%). This variability indicates substantial differences across studies, likely due to variations in methodological design, participant characteristics, intervention types, or outcome measures. Nevertheless, the overall effect remained significant, reinforcing the general efficacy of psychological interventions—particularly CBT, family therapy, and executive function training. Similar results have been reported by Yue and Rich (2023) [63].

From a theoretical perspective, the findings can be interpreted within the framework of the I-PACE model (Interaction of Person–Affect–Cognition–Execution) [33,64], which proposes that addictive or problematic behaviors develop and persist through the dynamic interaction between predisposing personal factors (e.g., impulsivity or emotional vulnerability), affective and cognitive responses to specific stimuli, and executive control processes. Within this framework, CBT may exert its effectiveness through two primary mechanisms: first, by strengthening executive functions—particularly inhibitory control and self-regulation—via cognitive restructuring and behavioral training strategies; and second, by reducing sensitivity to reward cues associated with problematic behaviors through the modification of expectations and cognitive biases related to immediate gratification [55].

Taken together, these findings suggest that CBT may facilitate the functional modulation of frontostriatal circuits involved in impulse regulation and reward processing, thereby decreasing cue reactivity associated with problematic behaviors and enhancing cognitive control over such behaviors, in accordance with the principles of the I-PACE model.

This trend was also observed in non-RCTs, which demonstrated a slightly smaller pooled effect size (SMD = −1.13) and moderate heterogeneity (I^2^ = 56.72%), indicating more consistent results across studies, albeit with lower methodological rigor. These findings are consistent with previous research supporting the effectiveness of psychological interventions for non-substance-related addictive behaviors among adolescents [65,66].

Although the combined effect sizes observed (SMD = –1.53 for RCTs and SMD = –1.13 for non-RCTs) reflect substantial reductions in addictive symptomatology, it is essential to consider their clinical relevance in addition to statistical significance. These effects indicate meaningful improvements in ICT use regulation, emotional self-control, and overall well-being among adolescents. However, the limited number of studies incorporating medium- and long-term follow-ups restricts the ability to evaluate the durability of these benefits over time. Therefore, future research should adopt longitudinal designs to assess the stability of clinical improvements and their potential impact on functional domains such as academic performance, family relationships, and psychosocial adjustment.

In particular, CBT has demonstrated effectiveness in modifying dysfunctional cognitive patterns and enhancing self-regulation in ICT use, both in group-based and individual formats [67,68]. When combined with CBT, family-centered therapies have shown benefits in improving parental control and communication—key factors in preventing ICT addiction [69]. Moreover, therapeutic interventions targeting executive functions, specifically through the training of rash impulsivity and reward sensitivity, have collectively demonstrated improvements in inhibitory control over problematic behaviors and a reduction in the motivational tendency to approach ICT-related stimuli associated with reinforcement [55].

The meta-regression analyses identified key moderators influencing the effectiveness of interventions. Among RCTs, both longer duration and individual delivery formats were associated with greater reductions in problematic ICT use, consistent with evidence that sustained and personalized approaches foster stronger adherence and behavioral change. In contrast, cultural context did not significantly affect RCT outcomes, suggesting that the core cognitive-behavioral mechanisms underlying these interventions may be broadly applicable across cultures.

However, in non-RCTs, cultural context emerged as a significant moderator, with studies conducted in Eastern countries showing larger effects than those conducted in Western contexts. This may reflect cultural variations in family involvement, social cohesion, and attitudes toward technology use, underscoring the importance of culturally sensitive adaptations in the development and implementation of preventive and therapeutic programs [70].

The fourth objective—to generate recommendations for clinical practice—highlights the importance of early identification of psychological distress, family involvement, limiting screen time, and implementing psychoeducational programs that engage both adolescents and their families. These recommendations align with the findings of Qi and Yang (2024) [71], who reported an inverse relationship between digital resilience and technology-related stress in adolescents. Their study underscores the need for comprehensive interventions that address psychological, social, and contextual factors while promoting coping and self-regulation skills across family, school, and leisure environments.

Beyond the clinical implications, the findings of this study hold interdisciplinary relevance that extends beyond the field of psychology. Consistent with the digital wellbeing perspective proposed by Orben et al. (2019, *PNAS*) [72], the results support the need for collaborative frameworks integrating psychological research, educational policies, and technological regulation. Evidence of the effectiveness of cognitive-behavioral and family-based interventions can inform school programs promoting digital literacy and the prevention of problematic ICT use, as well as guide regulatory strategies aimed at the ethical design of digital platforms. From this standpoint, translating therapeutic principles into digital design could foster environments that promote self-regulation, resilience, and healthy social interaction among adolescents. Therefore, future research should move toward an integrated approach combining psychological evidence with public policy, design ethics, and digital health strategies to reduce the risks associated with intensive technology use and promote sustainable digital well-being.

However, several limitations should be acknowledged. First, the small number of studies included in the systematic review and meta-analysis limits the generalizability of the findings and may reduce the stability of the estimated effect sizes. Second, the asymmetry observed in the funnel plots suggests possible publication bias, as studies reporting null or non-significant effects appear to be missing. Although no formal statistical tests were performed due to the limited sample size, such bias could lead to an overestimation of the overall effect [71].

Another relevant limitation is the methodological heterogeneity among studies. Variations were identified in diagnostic criteria, assessment tools, duration and format of interventions, follow-up protocols, and outcome measures. These discrepancies hinder direct comparison across studies and may influence the observed effect sizes.

Despite the consistent direction of effects favoring psychological interventions, the certainty of the evidence was rated as *very low* according to the GRADE framework. This rating reflects the high risk of bias identified in most studies, the substantial statistical heterogeneity, and the possible presence of publication bias. Consequently, the estimates of effectiveness should be interpreted with caution and considered preliminary.

Future research should explore differential effects by gender, the moderating role of specific ICT types and sociocultural contexts, and the effectiveness of personalized, multidisciplinary therapeutic and preventive interventions that address the complexity of these addictive behaviors. Collaboration between mental health professionals, educators, and families will be essential to optimize prevention and therapeutic strategies for this at-risk group. It is recommended that future studies replicate interventions combining CBT with training focused on impulsive behavior, particularly regarding action tendencies towards stimuli conditioned by reinforcement and reward sensitivity. Moreover, future studies should aim to expand the empirical base through methodologically rigorous randomized controlled trials with more representative samples. These lines of inquiry will help identify the most effective components of interventions and tailor them to the specific needs of the adolescent population.

## 5. Conclusions

The findings of this systematic review and meta-analysis indicate that psychological therapeutic and preventive interventions, particularly CBT, are effective in reducing symptoms associated with Internet, video game, online gaming, and social media addiction in adolescents [73,74]. CBT has shown significant benefits in areas such as time management, reduction in subjective distress, improved self-regulation, and enhanced personal and social functioning, both in individual and group or family-based interventions [74]. Additionally, other approaches such as executive function training and family therapy have yielded promising results, although the current scientific evidence supporting these approaches remains limited [75,76].

In comparative terms, experimental groups exhibited significantly greater improvements than control groups, with large effect sizes observed in both RCTs (SMD = –1.53) and non-RCTs (SMD = –1.13). These results suggest that participation in structured programs based on CBT, family therapy, and executive function training produces a direct reduction in addictive symptomatology, whereas non-specialized interventions or the absence of treatment yield limited benefits.

The greater effectiveness observed in the intervention groups may be explained by psychological mechanisms described in the I-PACE model, including improvements in inhibitory control and emotional self-regulation, reductions in reward sensitivity associated with ICT use, and the restructuring of cognitive biases linked to immediate gratification. In family-based interventions, parental or caregiver involvement strengthens communication and parental monitoring, factors that enhance treatment adherence and prevent relapse.

The findings of this review have important translational implications for digital health policy and international research. First, evidence of the effectiveness of CBT-based psychological interventions supports their inclusion in school and community programs promoting digital literacy and preventing problematic ICT use. Second, the results contribute to elucidating transdiagnostic mechanisms—such as deficits in inhibitory control, increased reward sensitivity, and cognitive biases—that are shared across various behavioral addictions, consistent with the I-PACE model. Finally, integrating clinical evidence with educational policies and ethical digital design can inform global public health strategies aimed at promoting adolescent digital well-being.

Nonetheless, there remains a need to develop and validate specific protocols for addressing problematic social media use and to tailor interventions to the individual and contextual characteristics of adolescents. Therefore, both clinical practice and future research should adopt personalized and multidisciplinary approaches, integrating psychoeducation, family support, and the promotion of healthy digital habits. Such strategies aim to optimize the prevention and treatment of problematic ICT use among adolescents [65,77,78].

In summary, psychological interventions appear promising for reducing problematic ICT use among adolescents and young adults. However, according to the GRADE assessment, the overall certainty of the evidence remains very low. Future randomized controlled trials employing rigorous methodologies, larger and more diverse samples, and standardized outcome measures are needed to strengthen confidence in these findings and assess the long-term sustainability of treatment effects.

Longer and individually delivered interventions demonstrated greater effectiveness in reducing problematic ICT use, emphasizing the importance of sustained and personalized approaches. Cultural context influenced outcomes in non-RCTs, with stronger effects reported in Eastern settings, underscoring the necessity of culturally adapted programs. Overall, both methodological and contextual factors should be considered to optimize intervention design and implementation.

## Data Availability

The raw data supporting the conclusions of this article will be made available by the authors on request.

## Supplementary Materials

The following supporting information can be downloaded at: https://www.mdpi.com/article/10.3390/ijerph22101598/s1, Table S1: Characteristics of the included RCT studies for the treatment of ICT addiction; Table S2: Characteristics of the included Quasi-experimental non-RCT studies for the treatment of ICT addiction. Table S3: Risk of bias of the RCT and non-RCT studies included in the systematic review. Table S4: PRISMA 2020 checklist. Table S5. Summary of Findings (SoF).

## Abbreviations

The following abbreviations are used in this manuscript:

ICT	Information and Communication Technologies
WHO	World Health Organization
DSM-5	Diagnostic and Statistical Manual of Mental Disorders, Fifth Edition
PIU	Problematic Internet Use
BMHD	Bioecological Model of Human Development
IAM	Internet Addiction Model
I-PACE	An Interaction of Person-Affect-Cognition-Execution
UCJC	Universidad Camilo José Cela
IGD	Internet Gaming Disorder Multidisciplinary
EG	Experimental Group
CG	Control Group
CBT	Cognitive Behavioral Therapy
SMA	Social Media Addiction
DMUD	Digital media-use disorders
IUD	Internet Use Disorder
GAS	Game Addiction Scale; NODS-CLiP
IGD-20	Test: Internet Gaming Disorder Test
YSR/11–18	Youth Self-Report for Ages 11–18
CBCL/6–18	Child Behavior Checklist for Ages 6–18 Years
MACI	Expressed Concern Scales of the Millon Adolescent Clinical Inventory
TMMS-24	Trait Meta-Mood Scale
EHS	Escala de Habilidades Sociales
WATOCI	Satisfaction with the treatment. The Working Alliance Theory of Change Inventory
CSAS	Computerspielabhäng-igkeitsskala
SCL-90-R	Symptom Checklist-Revised
STAI	State-Trait Anxiety Index
DQVMIA	Diagnostic Questionnaires for Video Games, Mobile Phone or Internet Addiction
S-MASS	Social Media Addiction Screening Scale
GADIS-A	Gaming Disorder Scale for Adolescents
SOMEDIS-A	Social Media Disorder Scale for Adolescents
STREDIS-A	Streaming Disorder Scale for Adolescents
SDQ	Strength and Difficulties Questionnaire
PSQI	Pittsburgh Sleep Quality Index
ESS-CHAD	Epworth Sleepiness Scale-Children and Adolescents
PSS-10	Perceived Stress Scale
FCS	Family Communication Scale
MAAS-5	Mindfulness Attention Awareness Scale
CIAS	Chen Internet Addiction Scale
SAS	Self-rating Anxiety Scale
SDS	Self-rating Depression Scale
BISS-11	Barrat Impulsiveness;
fMRI	functional Magnetic Resonance Imaging
GD	Game-related outcome
C-RIGC	Revised Internet Gaming Cognition Scale
PHQ-9	Patient Health Questionnaire
GAD	Generalized Anxiety Disorder
