# Supplementary material for: Effectiveness of Psychological Treatments for Problematic Use of Internet, Video Games, Social Media and Instant Messaging: A Systematic Review and Meta-Analysis
Source: Int J Environ Res Public Health. 2025 Oct 21;22(10):1598. doi: 10.3390/ijerph22101598 (PMC12563152; doi:10.3390/ijerph22101598)
Supplement: Supplementary file 1 [file ijerph-22-01598-s001.zip › ijerph-3832912-supplementary_2nd proofed (1).pdf]

Table S1. Characteristics of the included RCT studies for the treatment of ICT addiction.

ID	First author (year) / Country	Type of addiction ICT / treatment / type of treatment	Sample size (range of years)	Outcome measures	Aims	Number of sessions
1	Ji & Wong (2023) / China [54]	Gaming disorder / integrated CBT + motivational approach	38 intervention + 39 controls students (aged 14-21)	GD symptoms, gaming motivation, depression, anxiety	Test ICBT effectiveness with motivational approach	8 sessions
2	Kochuchakkalackal & Reyes (2023) / Philippines [58]	Internet Gaming Disorder / Acceptance and Cognitive Restructuring Intervention Program (ACRIP)	15 intervention + 15 controls (aged 16-19)	IGDS9-SF, Ryff's Psychological Well-being Scales	Evaluate ACRIP's cross-cultural efficacy in reducing IGD symptoms and improving psychological well-being	16 sessions
3	Lindenberg et al. (2022) / Germany [52]	Internet Gaming disorder & internet use disorder / PROTECT CBT-based preventive group	85 intervention + 126 controls (aged 12–18)	CSAS scale, incidence of GD/IUD	Test PROTECT program effectiveness	4 sessions
4	Uysal & Balci (2018) / Turkey [53]	Internet addiction / Healthy Internet Use Program (school-based program)	41 intervention + 43 control students (aged 11–13)	Internet Addiction Scale	Evaluate effectiveness of a school-based program to reduce internet addiction	8 sessions
5	Zheng et al. (2022) / China [55]	Internet Gaming Disorder / combined behavioral intervention for reward sensitivity and rash impulsiveness	20 intervention RI + 20 intervention ApBM + 20 intervention combined + 20 controls (average 14.75 aged)	Online Game Addiction Scale (OGAS), Barratt Impulsiveness Scale, DASS-21	Compare effectiveness of combined vs. single behavioral interventions on IGD	15 sessions

* IGD: Internet Gaming Disorder; ICT: Information and Communication Technologies; EG: Experimental Group; CG: Control Group; CBT: Cognitive Behavioral Therapy; SMA: Social Media Addiction; DMUD: Digital media-use disorders; IUD: Internet Use Disorder; GAS: Game Addiction Scale; NODS-CLiP: Diagnostic Screen for Gambling Problems–Control, Lying, and Preoccupation; IGD-20 Test: Internet Gaming Disorder Test; YSR/11-18: Youth Self-Report for Ages 11–18; CBCL/6-18: Child Behavior Checklist for Ages

6–18 Years; MACI: Expressed Concern Scales of the Millon Adolescent Clinical Inventory; TMMS-24: Trait Meta-Mood Scale; EHS: Escala de Habilidades Sociales; WATOCI: Satisfaction with the treatment. The Working Alliance Theory of Change Inventory; CSAS: Computerspielabhängigkeitsskala; SCL-90-R: Symptom Checklist-Revised; STAI: State-Trait Anxiety Index; DQVMIA: Diagnostic Questionnaires for Video Games, Mobile Phone or Internet Addiction; S-MASS: Social Media Addiction Screening Scale; GADIS-A: Gaming Disorder Scale for Adolescents; SOMEDIS-A: Social Media Disorder Scale for Adolescents; STREDIS-A: Streaming Disorder Scale for Adolescents; SDQ: Strength and Difficulties Questionnaire; PSQI: Pittsburgh Sleep Quality Index; ESS-CHAD: Epworth Sleepiness Scale - Children and Adolescents; PSS-10: Perceived Stress Scale; FCS: Family Communication Scale; MAAS-5: Mindfulness Attention Awareness Scale; CIAS: Chen Internet Addiction Scale; SAS: Self-rating Anxiety Scale; SDS. Self-rating Depression Scale; BISS-11: Barrat Impulsiveness; fMRI: functional Magnetic Resonance Imaging; GD: Game-related outcomes; C-RIGC: Revised Internet Gaming Cognition Scale; PHQ-9: Patient Health Questionnaire; GAD: Generalized Anxiety Disorder. .

Table S2. Characteristics of the included Quasi-experimental non-RCT studies for the treatment of ICT addiction.

ID	First author (year) / Country	Type of addiction ICT / treatment / type of treatment	Sample size (range of years)	Outcome measures	Aims	Number of sessions
1	Kumkronglek et al. (2023) / Thailand [56]	Social media addiction / life skills enhancement program	24 intervention + 21 controls (aged 12–15)	Social Media Addiction Screening Scale (S-MASS), life skills test	Develop and assess life skills program for social media addiction	10 sessions
2	Pornnoppadol et al. (2020) / Thailand [57]	Internet Gaming Disorder / S-TRC, PMT-G, combined, vs psychoeducation	74 intervention + 30 controls (aged 13–17)	Game Addiction Screening Test (GAST), Game Addiction Quality of Life Scale (GAME-Q)	Compare psychosocial interventions (S-TRC, PMT-G, combined) for IGD	10 sessions
3	Torres-Rodríguez et al. (2018) / Spain [51]	Internet Gaming Disorder / PIPATIC psychotherapy vs CBT	17 intervention + 17 controls (aged 12–18)	Ability to stop gaming, Self-awareness of engagement in gaming, IGD-20 Test, YSR/11-18, CBCL/6-18, MACI, TMMS-24, EHS, WATOCI	Evaluate PIPATIC effectiveness compared to standard CBT	22 sessions
4	Yang & Kim (2018) / South Korea [59]	Internet addiction / self-regulatory efficacy improvement program led by school nurses	38 intervention + 41 control students (aged 13–15)	Self-Control Scale, Self-Efficacy Scale, Internet Addiction Proneness Scale	Assess effects of self-regulation/self-efficacy program on internet addiction prevention	10 sessions

* IGD: Internet Gaming Disorder; ICT: Information and Communication Technologies; EG: Experimental Group; CG: Control Group; CBT: Cognitive Behavioral Therapy; SMA: Social Media Addiction; DMUD: Digital media-use disorders; IUD: Internet Use Disorder; GAS: Game Addiction Scale; NODS-CLiP: Diagnostic Screen for Gambling Problems–Control, Lying, and Preoccupation; IGD-20 Test: Internet Gaming Disorder Test; YSR/11-18: Youth Self-Report for Ages 11–18; CBCL/6-18: Child Behavior Checklist for Ages 6–18 Years; MACI: Expressed Concern Scales of the Millon Adolescent Clinical Inventory; TMMS-24: Trait Meta-Mood Scale; EHS: Escala de Habilidades Sociales; WATOCI: Satisfaction with the treatment. The Working Alliance Theory of Change Inventory; CSAS: Computerspielabhängigkeitsskala; SCL-90-R: Symptom Checklist-Revised; STAI: State-Trait Anxiety Index; DQVMIA: Diagnostic Questionnaires for Video Games, Mobile Phone or Internet Addiction; S-MASS: Social Media Addiction Screening Scale; GADIS-A: Gaming Disorder Scale for Adolescents; SOMEDIS-A: Social Media Disorder Scale for Adolescents; STREDIS-A: Streaming Disorder Scale for Adolescents; SDQ: Strength and Difficulties Questionnaire; PSQI: Pittsburgh Sleep Quality Index; ESS-CHAD: Epworth Sleepiness Scale - Children and Adolescents; PSS-10: Perceived Stress Scale; FCS: Family Communication Scale; MAAS-5: Mindfulness Attention Awareness Scale; CIAS: Chen Internet Addiction Scale; SAS: Self-rating Anxiety Scale; SDS. Self-rating Depression Scale; BISS-11: Barrat Impulsiveness; fMRI: functional Magnetic Resonance Imaging; GD: Game-related outcomes; C-RIGC: Revised Internet Gaming Cognition Scale; PHQ-9: Patient Health Questionnaire; GAD: Generalized Anxiety Disorder.

Table S3. Risk of bias of the RCT and non-RCT studies included in the systematic review.

Study	Type of study	Domain 1		Domain 2		Domain 3	Domain 4	Domain 5	Quality
Lindenberg et al. (2022) [52]	RCT	+		?		-	+	+	+[a]
Kochuchakkalackal et al. (2024) [58]	RCT	?		?		+	+	?	?[a]
Ji et al. (2023) [54]	RCT	+		?		+	?	?	?[a]
Uysal et al. (2018) [53]	RCT	?		?		-	+	?	?[a]
Zheng et al. (2022) [55]	RCT	?		?		+	+	?	?[a]
		**Item 1**	**Item 2**	**Item 3**	**Item 4**	**Item 5**	**Item 6**	**Item 7**	
Kumkronglek et al. (2023) [56]	Non- RCT	?	?	+	+	+	?	?	?[b]
Pornnoppadol et al. (2020) [57]	Non- RCT	?	+	+	+	+	?	+	+[b]
Torres-Rodríguez et al. (2018) [51]	Non- RCT	+	+	+	?	+	?	?	?[b]
Yang et al. (2018) [59]	Non- RCT	?	+	+	+	+	?	+	+[b]

Note. + = Low risk of bias; ?= Some risk of bias; - = High risk of bias.
a = quality assessment by Revised Cochrane risk-of-bias tool for randomized trials (RoB2) (Higgins et al., 2019)[1].
b = quality assessment by ROBINS-E tool (Higgins et al., 2024)[2].

Table S4. PRISMA 2020 Checklist.

Section and Topic	Item #	Checklist item	Location where item is reported
**TITLE:** Effectiveness of Psychological Treatments for Problematic Use of Internet, Video Games, Social Media and Instant Messaging: A Systematic Review and Meta-analysis.			
Title	1	Identify the report as a systematic review.	First page
**ABSTRACT**			
Abstract	2	See the PRISMA 2020 for Abstracts checklist.	1 (Abstract section)
**INTRODUCTION**			
Rationale	3	Describe the rationale for the review in the context of existing knowledge.	Introduction, paragraphs 1–6
Objectives	4	Provide an explicit statement of the objective(s) or question(s) the review addresses.	Introduction, final paragraph (specific objectives listed)
**METHODS**			
Eligibility criteria	5	Specify the inclusion and exclusion criteria for the review and how studies were grouped for the syntheses.	Materials and Methods, section 2.2 (Study Selection…)
Information sources	6	Specify all databases, registers, websites, organisations, reference lists and other sources searched or consulted to identify studies. Specify the date when each source was last searched or consulted.	Materials and Methods, section 2.1 (Search Strategy)
Search strategy	7	Present the full search strategies for all databases, registers and websites, including any filters and limits used.	Materials and Methods, section 2.1 (full terms and Boolean operators)
Selection process	8	Specify the methods used to decide whether a study met the inclusion criteria of the review, including how many reviewers screened each record and each report retrieved, whether they worked independently, and if applicable, details of automation tools used in the process.	Materials and Methods, section 2.2 (Study Selection)
Data collection process	9	Specify the methods used to collect data from reports, including how many reviewers collected data from each report, whether they worked independently, any processes for obtaining or confirming data from study investigators, and if applicable, details of automation tools used in the process.	Materials and Methods, section 2.3 (Data Synthesis and Statistical Analysis: narrative synthesis and extraction)
Data items	10a	List and define all outcomes for which data were sought. Specify whether all results that were compatible with each outcome domain in each study were sought (e.g. for all measures, time points, analyses), and if not, the methods used to decide which results to collect.	Materials and Methods, section 2.3 (main outcome = effect of CBT on problematic ICT use)
	10b	List and define all other variables for which data were sought (e.g. participant and intervention characteristics, funding sources). Describe any assumptions made about any missing or unclear information.	Results, section 3.1 (additional variables such as impulsivity, personality patterns, emotional symptoms, etc.)

Section and Topic	Item #	Checklist item	Location where item is reported
Study risk of bias assessment	11	Specify the methods used to assess risk of bias in the included studies, including details of the tool(s) used, how many reviewers assessed each study and whether they worked independently, and if applicable, details of automation tools used in the process.	Materials and Methods, section 2.2 (Quality and Risk of Bias Assessment)
Effect measures	12	Specify for each outcome the effect measure(s) (e.g. risk ratio, mean difference) used in the synthesis or presentation of results.	Materials and Methods, section 2.3 (SMDs, heterogeneity statistics)
Synthesis methods	13a	Describe the processes used to decide which studies were eligible for each synthesis (e.g. tabulating the study intervention characteristics and comparing against the planned groups for each synthesis (item #5)).	Materials and Methods, section 2.2–2.3
	13b	Describe any methods required to prepare the data for presentation or synthesis, such as handling of missing summary statistics, or data conversions.	Materials and Methods, section 2.3 (separate analyses for RCTs and non-RCTs, random-effects model)
	13c	Describe any methods used to tabulate or visually display results of individual studies and syntheses.	Results, Figures 2–5 (forest plots, funnel plots)
	13d	Describe any methods used to synthesize results and provide a rationale for the choice(s). If meta-analysis was performed, describe the model(s), method(s) to identify the presence and extent of statistical heterogeneity, and software package(s) used.	Materials and Methods, section 2.3
	13e	Describe any methods used to explore possible causes of heterogeneity among study results (e.g. subgroup analysis, meta-regression).	Results, section 3.3 (I^2, tau^2, Q tests)
	13f	Describe any sensitivity analyses conducted to assess robustness of the synthesized results.	Not explicitly reported
Reporting bias assessment	14	Describe any methods used to assess risk of bias due to missing results in a synthesis (arising from reporting biases).	Results, section 3.3 (funnel plot description)
Certainty assessment	15	Describe any methods used to assess certainty (or confidence) in the body of evidence for an outcome.	Not explicitly reported
**RESULTS**			
Study selection	16a	Describe the results of the search and selection process, from the number of records identified in the search to the number of studies included in the review, ideally using a flow diagram.	Results, section 3.1; Figure 1 (PRISMA flow diagram)
	16b	Cite studies that might appear to meet the inclusion criteria, but which were excluded, and explain why they were excluded.	Not explicitly listed (no table of excluded studies)
Study characteristics	17	Cite each included study and present its characteristics.	Results, section 3.1; Supplementary Tables S1 & S2
Risk of bias in studies	18	Present assessments of risk of bias for each included study.	Results, section 3.2; Supplementary Table S3
Results of individual studies	19	For all outcomes, present, for each study: (a) summary statistics for each group (where appropriate) and (b) an effect estimate and its precision (e.g. confidence/credible interval), ideally using structured tables or plots.	Results, section 3.3 (effect sizes per study, forest plots)

Section and Topic	Item #	Checklist item	Location where item is reported
Results of syntheses	20a	For each synthesis, briefly summarise the characteristics and risk of bias among contributing studies.	Results, sections 3.1–3.2
	20b	Present results of all statistical syntheses conducted. If meta-analysis was done, present for each the summary estimate and its precision (e.g. confidence/credible interval) and measures of statistical heterogeneity. If comparing groups, describe the direction of the effect.	Results, section 3.3; Figures 2 & 4
	20c	Present results of all investigations of possible causes of heterogeneity among study results.	Results, section 3.3
	20d	Present results of all sensitivity analyses conducted to assess the robustness of the synthesized results.	Not reported
Reporting biases	21	Present assessments of risk of bias due to missing results (arising from reporting biases) for each synthesis assessed.	Results, section 3.3 (funnel plot commentary)
Certainty of evidence	22	Present assessments of certainty (or confidence) in the body of evidence for each outcome assessed.	Not reported
**DISCUSSION**			
Discussion	23a	Provide a general interpretation of the results in the context of other evidence.	Discussion, paragraphs 1–4
	23b	Discuss any limitations of the evidence included in the review.	Discussion, paragraph 8
	23c	Discuss any limitations of the review processes used.	Discussion, paragraph 8
	23d	Discuss implications of the results for practice, policy, and future research.	Discussion, paragraphs 9–10
**OTHER INFORMATION**			
Registration and protocol	24a	Provide registration information for the review, including register name and registration number, or state that the review was not registered.	Materials and Methods, section 2 (PROSPERO CRD420251029371)
	24b	Indicate where the review protocol can be accessed, or state that a protocol was not prepared.	Not reported (only registration number given)
	24c	Describe and explain any amendments to information provided at registration or in the protocol.	Not reported
Support	25	Describe sources of financial or non-financial support for the review, and the role of the funders or sponsors in the review.	Funding statement (“This research received no external funding.”)
Competing interests	26	Declare any competing interests of review authors.	Conflicts of Interest section
Availability of data, code and other materials	27	Report which of the following are publicly available and where they can be found: template data collection forms; data extracted from included studies; data used for all analyses; analytic code; any other materials used in the review.	Data Availability Statement (“available upon request”)

From: Page MJ, McKenzie JE, Bossuyt PM, Boutron I, Hoffmann TC, Mulrow CD, et al. The PRISMA 2020 statement: an updated guideline for reporting systematic reviews. BMJ 2021;372:n71. doi: 10.1136/bmj.n71. This work is licensed under CC BY 4.0. To view a copy of this license, visit https://creativecommons.org/licenses/by/4.0/ [3]

Table S5. Summary of Findings (SoF).

Outcome	Control group	Intervention effect (95% CI)	Effect measure	Participants (studies)	Certainty of evidence (GRADE)	Comments
Problematic/addictive use symptoms (RCTs, post-treatment)	—	SMD = −1.53 (−2.91 to −0.14)	Standardised mean difference (REML)	482 participants (7 estimates, 5 RCTs)	⊕◯◯◯ Very low	High risk of bias in ≈80 % of RCTs; extremely high heterogeneity (I^2 = 98.2 %); suspected publication bias (funnel-plot asymmetry).
Problematic/addictive use symptoms (non-RCTs, post-treatment)	—	SMD = −1.13 (−1.59 to −0.67)	Standardised mean difference (REML)	262 participants (4 studies)	⊕◯◯◯ Very low	Non-randomised design (confounding likely); moderate heterogeneity (I^2 = 56.7 %); possible publication bias.
Serious adverse events	—	Not reported	—	—	Not assessed	Adverse events were not systematically reported across studies.
Follow-up outcomes (≥ 3–6 months)	—	Insufficient data	—	—	Not assessed	Most studies reported only immediate post-treatment effects.

Note: Population: adolescents and young adults (11–21 years) with problematic or addictive use of digital technologies (PIU, IGD, SMA); Intervention: psychological therapies (e.g. cognitive-behavioural, family, executive-function training, integrative approaches); Comparator: treatment-as-usual (TAU), waiting list, or active controls; Outcome: post-treatment change in problematic/addictive use symptoms.

Explanatory notes

a) Risk of bias (−1): majority of RCTs rated as "some concerns"; non-RCTs inherently at high risk of confounding. b) Inconsistency (−2 RCTs, −1 non-RCTs): very serious for RCTs (I^2 = 98.2 %); serious for non-RCTs (I^2 = 56.7 %). c) Imprecision (−1 RCTs): wide 95 % CI including effects from minimal to very large; sample sizes small. d) Publication bias (−1): funnel-plot asymmetry indicates possible missing small studies with null effects.

Summary statement

Psychological interventions probably reduce symptoms of problematic technology use at post-treatment; however, the certainty of evidence is very low due to high risk of bias, substantial heterogeneity, and possible publication bias. Further high-quality randomised controlled trials with standardised outcome measures and follow-up assessments are warranted.

Reference Section

1. Higgins J.P.T.; López-López J.A.; Becker B.J.; Davies S.R.; Dawson S.; Grimshaw J.M.; McGuinness L.A.; Moore T.H.M.; Rehfuess E.A.; Thomas J.; et al. Synthesising quantitative evidence in systematic reviews of complex health interventions. *BMJ Glob Health*. **2019**, 4(Suppl 1):e000858. https://doi.org/10.1136/bmjgh-2018-000858
2. Higgins, J.P.T.; Thomas, J.; Chandler, J.; Cumpston, M.; Li, T.; Page, M.J.; Welch, V.A. (Eds.) *Cochrane Handbook for Systematic Reviews of Interventions version 6.5 (updated August 2024)*; Cochrane: Prague, Czech Republic, 2024. Available online: www.cochrane.org/handbook (accessed on 10 March 2025).
3. Page M.J.; McKenzie J.E.; Bossuyt P.M.;, Boutron I.; Hoffmann T.C.; Mulrow C.D.; Shamseer L.; Tetzlaff J.M.; Akl E.A.; Brennan S.E.; et. al. The PRISMA 2020 statement: an updated guideline for reporting systematic reviews. *BMJ* **2021**, 372:n71. doi: 10.1136/bmj.n71.
